# A tutorial review of stereoretentive olefin metathesis based on ruthenium dithiolate catalysts

**DOI:** 10.3762/bjoc.14.279

**Published:** 2018-12-07

**Authors:** Daniel S Müller, Olivier Baslé, Marc Mauduit

**Affiliations:** 1 Univ. Rennes, Ecole Nationale Supérieure de Chimie de Rennes, CNRS, ISCR – UMR 6226, F-35000 Rennes, France

**Keywords:** catalysis, olefin metathesis, ruthenium, stereoretentive

## Abstract

Stereoretentive olefin metathesis based on ruthenium dithiolate complexes has become a very active field of research within the past years. This unique catalyst class is able to kinetically produce both *Z*- and *E*-alkenes in high stereochemical purity (typically >95:5) starting from stereochemically pure *Z*- or *E*-alkenes. The aim of this tutorial review is to organize the reported information concerning ruthenium dithiolate catalysts in a logic manner, thus providing an "operators handbook" for chemists who wish to apply this methodology in synthesis.

## Review

### 1 Catalyst discovery and structure optimization from 2013–2018

**Scheme 1:**
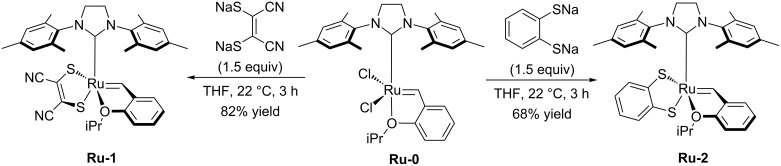
Synthesis of first Ru-dithiolate metathesis catalysts.

In stereoretentive metathesis the stereochemistry of the starting material is retained throughout the reaction: *Z-*alkenes starting materials lead to *Z*-alkene products and *E*-alkene starting materials lead to *E*-alkene products [1]. The first ruthenium dithiolate catalysts **Ru-1** and **Ru-2** were reported by Hoveyda in 2013 [2]. **Ru-1** and **Ru-2** were synthesized in one step from the commercially available Hoveyda–Grubbs catalyst **Ru-0** and the corresponding disodium dithiolate salts (Scheme 1).

**Figure 1:**
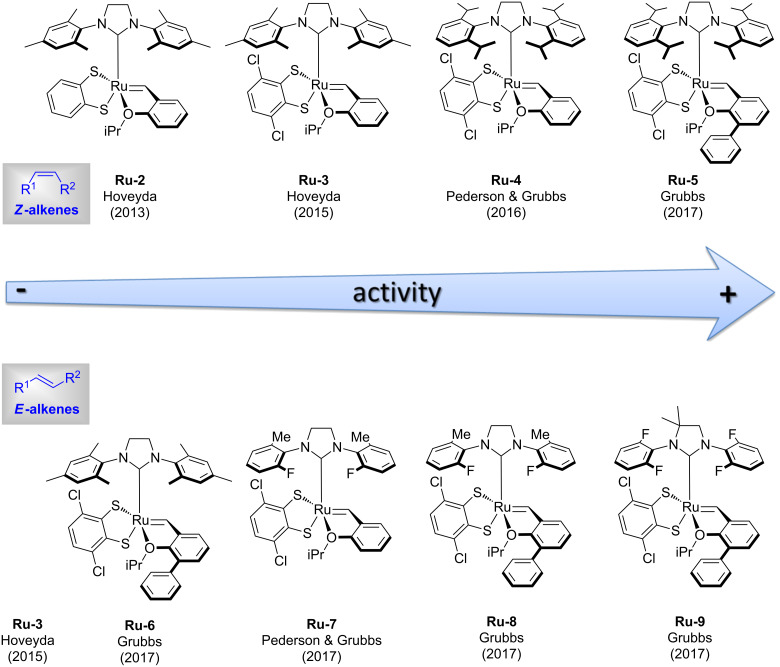
Most efficient Ru-dithiolate catalysts for stereoretentive olefin metathesis with *Z*- and *E*-alkenes as starting materials (activity increases from left to right).

Initially, Hoveyda described the complexes **Ru-1** and **Ru-2** as *Z*-selective catalysts [2]. However, subsequent studies by Pederson and the Grubbs group showed that Ru dithiolate catalysts are not stereoselective but stereoretentive catalysts [3]. Given the significant difference in geometry of *Z*- and *E*-alkenes it is obvious that each type of alkene requires a different catalyst (Figure 1). In both the *Z*- and *E*-stereoretentive processes, **Ru-3** introduced by Hoveyda in 2015 [4] showed moderate to good catalytic activity and can therefore be considered as a relatively general catalyst (Figure 1). In 2016 Pederson and Grubbs reported SIPr-based catalyst **Ru-4** with increased catalytic activity for Z-alkenes (Figure 1) [3]. Further improvement was made by the synthesis of **Ru-5** bearing the Blechert ligand (2-isopropoxy-3-phenylbenzylidene) which is well known to lead to faster initiating Hoveyda-type ruthenium metathesis catalysts [5–6]. The same researchers also found the Blechert modification to significantly improve stereoretentive reactions with *E*-alkenes (**Ru-6**) [6]. Furthermore, Pederson and Grubbs also demonstrated that diminishing the size of the *ortho* substitutents of the *N*-aryl groups of the NHC-ligand increased the efficiency for stereoretentive metathesis with *E*-alkenes (**Ru-7** [3], **Ru-8** [6], and **Ru-9** [6]). It should be noticed that the catalyst ranking shown in Figure 1 only applies to 1,2-disubstituted alkenes. Trisubstituted alkenes react very sluggishly and usually work only with catalysts that are efficient for *E*-alkenes (vide infra). Finally, it should be noted, that the precursors of catalysts **Ru-5** to **Ru-9** are not commercially available which limits their practicality [7].

**Figure 2:**
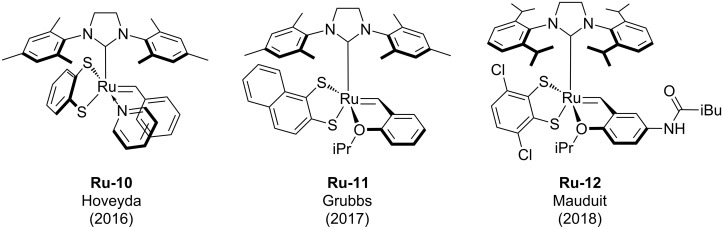
Selected examples of sterically or electronically modified ruthenium dithiolate complexes.

Other attempts to improve the efficiency of dithiolate catalysts by steric and electronic variation of the Ru-dithiolate complexes were reported by several research groups (Figure 2). Hoveyda and co-workers studied a series of catecholate, mercaptophenolate and catecholthiolate catalysts (e.g., **Ru-10**) [8–10]. Variation of sterically demanding catecholthiolate ligands was reported by Grubbs in 2017 (e.g., **Ru-11**) [11]. In 2018 our group reported a series of electronically and sterically activated dithiolate ruthenium catalysts (e.g., **Ru-12**) [12]. However, none of these studies identified more efficient or practical catalysts compared to the ones shown in Figure 1.

### 2 Mechanistic models

**Figure 3:**
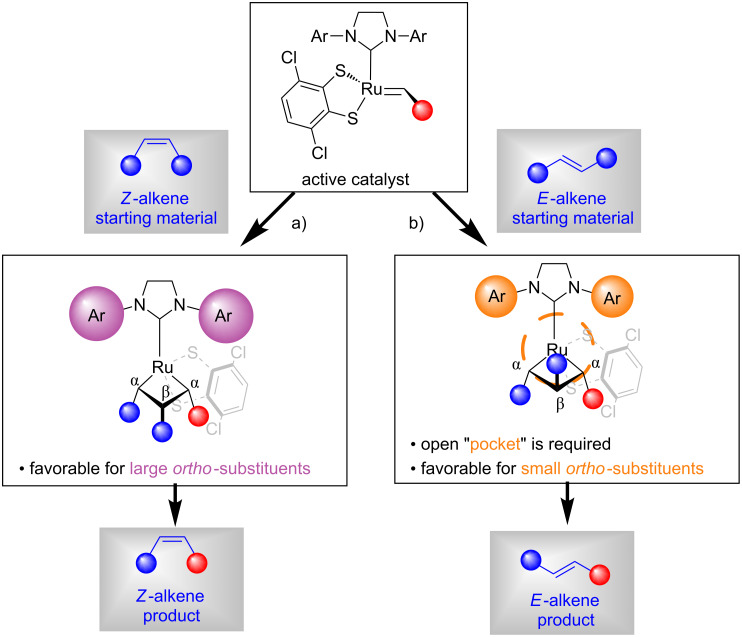
Model for stereoretentive metathesis proposed by Pederson and Grubbs [3].

The activity of the various catalysts vis-à-vis *Z*- or *E*-alkenes is best understood by a mechanistic model originally proposed by Pederson and Grubbs (Figure 3) [3]. A comprehensive computational study by Liu and Houk further validated this model, however, invoking distortion of the NHC ligand towards the dithiolate ligand as origin of the open pocket [13].

The proposed model assumes a side-bound mechanism, which results in a metallacycle perpendicular to the NHC ligand. To avoid steric repulsions, the substituents at the α-positions of the metallacycle point away from the *N*-aryl groups of the NHC-ligands. In contrast, the substituents at the β-position can point up or down. For the reaction with *Z*-alkenes (Figure 3a), the substituent at the β-position has to point down thus creating a new *Z*-alkene with the residing substituent shown in red. It is obvious, that blocking the open space above the β-position of the metallacycle with a very bulky SIPr-NHC ligand (e.g., **Ru-4** and **Ru-5**) has a positive effect on reactions with *Z*-alkenes. Reactions with *E*-alkenes follow the same logic (Figure 3b), however, placing the substituent on the β-position above the plane of the metallacycle pointing towards the NHC ligand. Therefore, it is critical to keep the "pocket" above the β-position open to accommodate the substituent of the incoming alkene. This explains why the smaller 2-fluoro-6-methylphenyl substituent on the NHC ligand (**Ru-7**) leads to higher activity for reactions with *E*-alkenes compared to its *N*-mesityl-substituted congener **Ru-3**. The same applies for trisubstituted alkenes where one substituent is forced into the open "pocket" in the β-position. Therefore, trisubstituted alkenes work best with the same catalysts used for *E*-alkenes (e.g., **Ru-7**, **Ru-8** and **Ru-9**).

### 3 Kinetic studies

**Figure 4:**
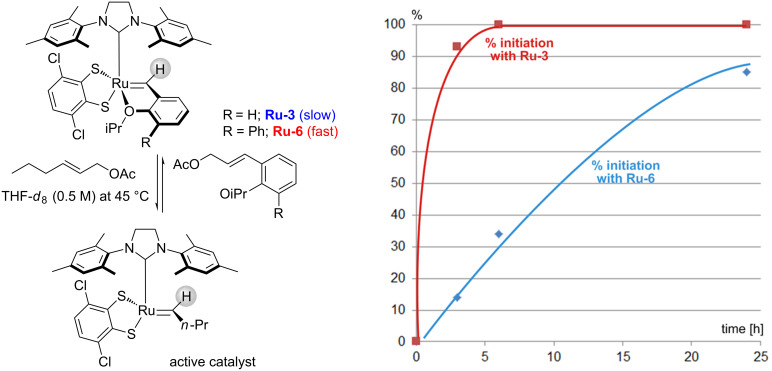
Decrease in the benzylidene signal over time upon reaction with (*E*)*-*2-hexenyl acetate.

Grubbs studied the kinetic behavior of several Ru-dithiolate catalysts [6,14–15]. In a typical study the disappearance of the benzylidene proton of the ruthenium complex with time is recorded. The disappearance is attributed to the formation of the active catalyst without considering competitive degradation of the catalyst. Figure 4 shows the percentage of consumed precursor complexes **Ru-3** and **Ru-6** for the reaction with (*E*)*-*2-hexenyl acetate within 24 hours [6]. The Blechert modification (**Ru-6**) initiates much faster with (*E*)*-*2-hexenyl acetate compared to the parent catalyst **Ru-3**.

### 4 Selected applications

**Scheme 2:**
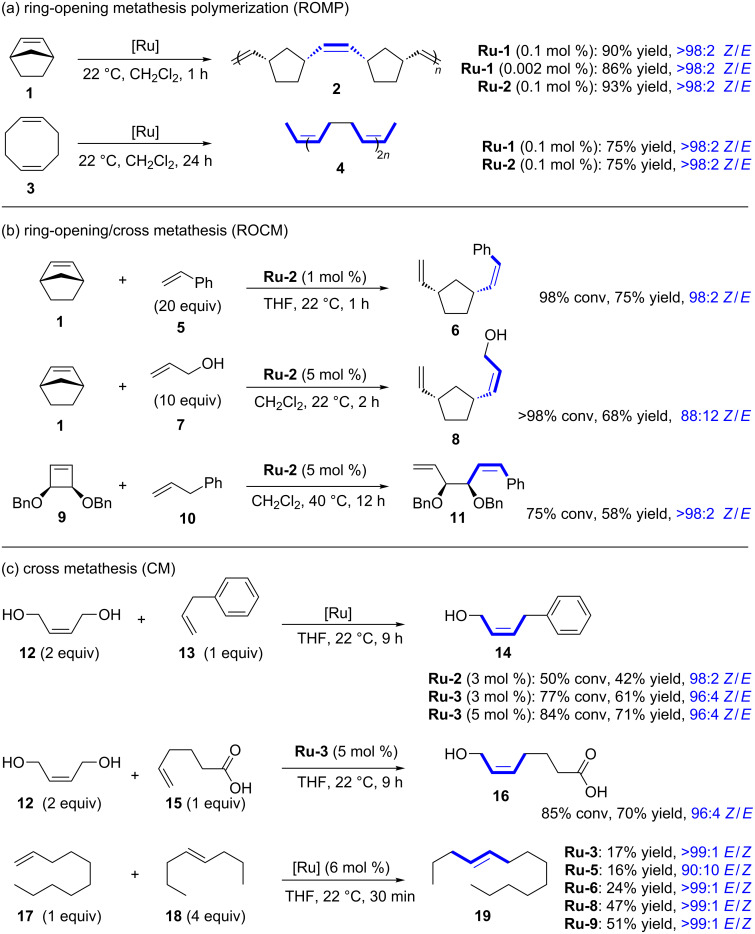
Selected applications, part 1.

**Scheme 3:**
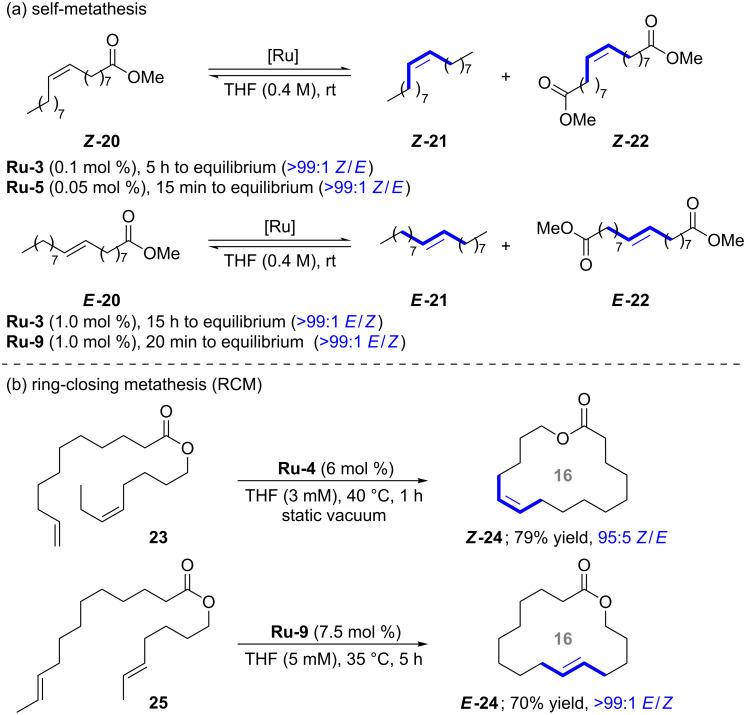
Selected applications, part 2.

The synthetic usefulness of ruthenium dithiolate catalysts was demonstrated in numerous synthetic applications such as ring-opening metathesis polymerization (ROMP), ring-opening/cross metathesis (ROCM), cross metathesis (CM), self-metathesis and ring-closing metathesis (RCM) reactions. Scheme 2 and Scheme 3 display selected examples for each of these reactions [1]. ROMP is one of the most facile metathesis reactions, thus allowing for very low catalyst loadings (Scheme 2a). Both catalysts **Ru-1** and **Ru-2** achieved excellent selectivities and good yields for the ROMP of norbornene (**1**) with catalyst loadings as low as 20 ppm (Scheme 2a) [2]. The ROMP of cyclooctadiene **3** was equally efficient with catalysts **Ru-1** and **Ru-2** [2]. It should be noted that the ROMP of norbornadiene was also investigated by Hoveyda [16]. A highly syndiotactic polymer was obtained by fine tuning of the steric and electronic characteristics of the catalyst (not depicted in this review) [16]. ROCM reactions of norbornene (**1**) with styrene (**5**) could be carried out with only one mole percent of catalyst loading Scheme 2b) [2]. Allylic alcohol (**7**) reacted cleanly with norbornene (**1**), albeit with lower stereoretention (**8**; 88:12 *Z*/*E*) [17]. Cyclobutenes (e.g., **9**) and cyclopropenes also delivered the corresponding products with good yields and excellent selectivity (Scheme 2b) [17]. It should also be noted that very recently Grubbs and Choi employed **Ru-3** for highly β-selective cyclopolymerization (not depicted in this review) [18]. Cross metathesis with *cis*-butendiol **12** was extensively explored by Hoveyda (Scheme 2c) [4]. The synthesis of *Z*-configured allylic alcohols is particularly attractive from the synthetic point of view. Allylic alcohols are highly versatile entities in organic chemistry and serve as starting materials in a multitude of reactions such as allylic substitutions [19]. Another advantage of this particular cross metathesis is that stereochemically pure *cis*-butenediol is commercially available and very inexpensive (≈40 €/500 mL) [20]. Catalyst loadings of 3 to 5 mol % are typically required to obtain useful yields of the corresponding allylic alcohols. The cross metathesis with carboxylic acid **15** is particularly noteworthy as cyclometallated *Z*-selective ruthenium catalysts are inefficient in the presence of acidic functional groups [4]. More recently, Grubbs reported the cross metathesis of 1-decene (**17**) and (*E*)*-*4-octene (**18**) [6]. The results obtained follow the ranking displayed in Figure 1 concerning the catalyst efficiency for reactions with *E*-alkenes. In accordance with the proposed model by Pederson and Grubbs (Figure 3), sterically demanding catalyst **Ru-5** afforded a 90:10 *E*/*Z* mixture indicating severe steric interaction between the SIPr-NHC ligand and the β-substituent of the *E*-alkene. The most productive catalysts for the cross metathesis with ***E***-**18** are those with small aryl substituents on the NHC moieties (**Ru-8** and **Ru-9**).

The self-metathesis of (*Z*)- and (*E*)-methyl 9-octadecenoate (**20**) was studied by Grubbs in 2017 (Scheme 3a) [6]. The efficiency of the catalysts follows the common trend displayed in Figure 1. Catalyst **Ru-5** achieved an equilibrium with perfect selectivity at only 500 ppm of catalyst loading within 15 minutes in contrast to parent catalyst **Ru-3** that required 5 hours at higher catalyst loading. *E*-Alkenes react more sluggishly, even optimized catalyst **Ru-9** required 1 mole percent of catalyst loading to achieve equilibirium within 20 minutes. Grubbs also studied the stereoretentive RCM reaction for the synthesis of *Z*- and *E*-configured macrocycles (e.g., **24**) [14–15]. As predicted from the working model, bulky catalyst **Ru-4** performed very well for the RCM reaction with *Z*-alkene **23**, whereas the smaller catalyst **Ru-9** performed best for *E*-alkene **25**.

**Figure 5:**
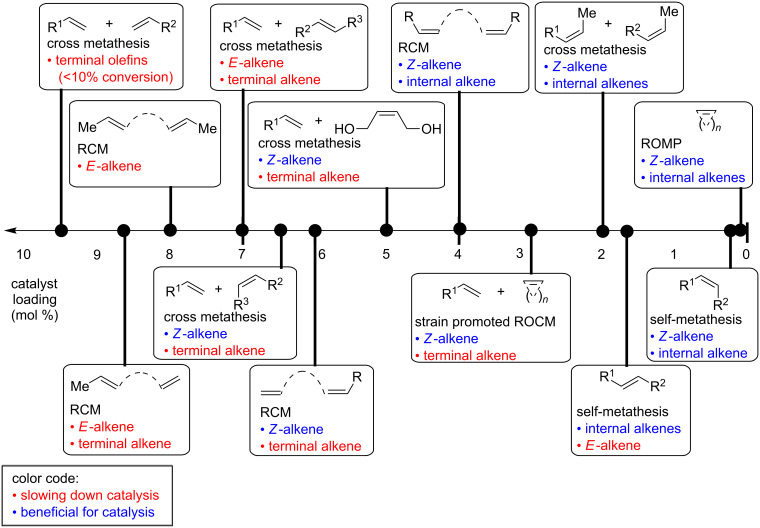
Catalyst loading required for different types of metathesis reactions.

According to the literature Figure 5 summarizes the approximate catalyst loadings required for each type of reaction reported with dithiolate catalysts. The first determining factor concerning the catalyst loadings is the configuration of the alkene: *Z*-alkenes react faster than *E*-alkenes and therefore require a lower catalyst loading (Figure 5). This can be easily understood by the mechanistic model proposed by Pederson and Grubbs (Figure 3). *Z*-Alkenes can easily approach to the catalyst via the widely open space underneath the metallacycle. In contrast, *E*-alkenes need to approach the catalyst in a way that the substituent above the metallacycle fits into the small open pocket; this is a less likely and slower process.

A second and even more important factor is the presence of terminal alkenes. Terminal alkenes are known to lead to catalyst degradation and therefore substrates containing terminal alkenes require high catalyst loading (see next section for details).

### 5 Catalyst stability

**Scheme 4:**
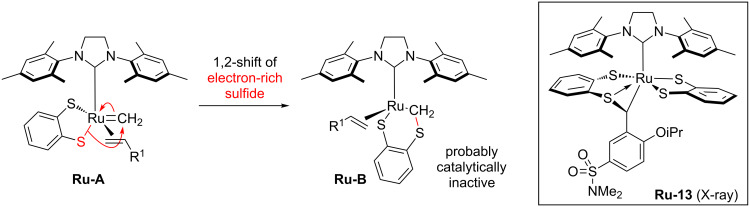
Proposed catalyst decomposition pathway occurring via attack of the electron-rich sulfide into methylidene ruthenium complex.

Hoveyda proposed that the catalyst degradation in the presence of terminal olefins is due to the generation of unstable methylidene-ruthenium species (Scheme 4) [4]. Terminal olefins inevitably produce ethylene which leads to the formation of methylidene-ruthenium species **Ru-A** (Scheme 4). Once complex **Ru-A** is formed, it is prone to be attacked by the electron-rich sulfide ligand positioned opposite to the NHC ligand (*trans*-influence). This 1,2-sulfide shift generates a new ruthenium complex **Ru-B** which is probably catalytically inactive.

This assumption is supported by the isolation of ruthenium complex **Ru-13** which was formed by nucleophilic attack of a sulfide ligand onto the electron-poor benzylidene ligand [4]. Hoveyda reasoned that replacing the thiocatecholate ligand (**Ru-2**) by an electron-deficient dichloro catecholthiolate (**Ru-3**) should render the sulfide ligand less nucleophilic and therefore less prone for nucleophilic attack. This hypothesis gained credence by increased isolated yield for the cross metathesis of allylbenzene with *cis*-butenediol: **Ru-2** (42% yield) versus **Ru-3** (61%) yield (Scheme 2c) [4].

### 6 The in situ methylene capping strategy

**Scheme 5:**
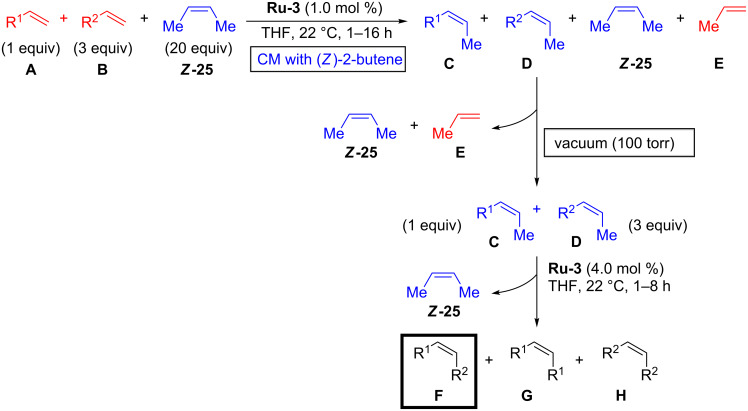
In situ methylene capping strategy for stereoretentive metathesis.

Experimental observations clearly indicate that terminal alkenes are detrimental for stereoretentive metathesis reactions with ruthenium dithiolate catalysts. In 2017 Hoveyda proposed the in situ methylene capping strategy as a solution to this problem [21]. The trick is to transform in situ the terminal olefins **A** and **B** into methylene capped olefins **C** and **D** by applying a large excess of (*Z*)-2-butene (***Z-*25**, Scheme 5). (*Z*)-2-butene (***Z-*25**) and propene **E** are then removed in vacuo (100 Torr) and a new portion of catalyst is added for the cross metathesis of **C** and **D** to give desired product **F** with excellent stereoisomeric purity along with side products **G** and **H** which require chromatographic removal. A major drawback of this strategy is that (*Z*)*-*2-butene (***Z-*25**) is not commercially available in many countries (e.g., in Europe).

#### Selected applications of the in situ methylene capping strategy

**Scheme 6:**
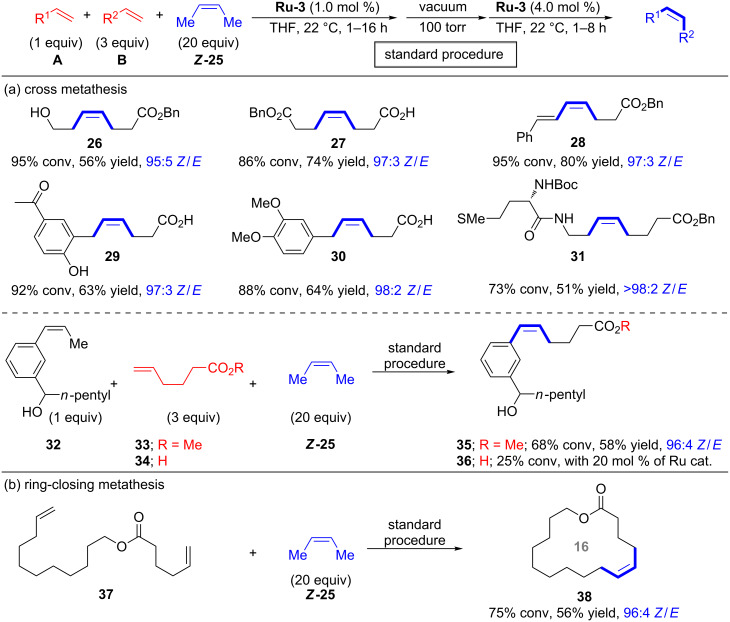
Stereoretentive cross-metathesis with (*Z*)-butene (***Z*-25**) as in situ methylene capping agent; selected applications.

Hoveyda and co-workers first applied the methylene capping strategy to cross-metathesis reactions (Scheme 6a) [21]. Almost 20 examples were isolated in modest to good yields and with excellent stereoisomeric purity. To assure high conversion in cross-metathesis reactions a 1:3 ratio of **A**/**B** was applied. Practical limitations are that **A** and **B** have to be of significantly different polarity for easy column chromatographic separation and that sterically hindered olefins are not tolerated. For some alkenes, e.g., styrenes, the homodimerization is too fast leading to stilbene formation. Replacing styrenes by (*Z*)-β-methylstyrenes (e.g., **32**) allowed for successful reactions with methyl ester **33** (Scheme 6a). Hoveyda noted that carboxylic acids (e.g., **34**) are not suitable cross-metathesis partners for (*Z*)-β-methylstyrenes. Hoveyda reasoned that with the sluggishly reacting styrene **32** the protonation and loss of the catechothiolate ligand by Brønsted acid **34** is a faster process leading to catalyst degradation. It should be noted that stereoretentive CM and RCM with (*E*)-2-butene (***E*-25**) as capping reagent were also reported, however, these reactions required a significantly higher catalyst loading (10.0–12.5 mol %) [21]. Macrocyclic ring-closing metathesis (RCM) with (*Z*)-butene (***Z*-25**) was also studied affording 14–21-membered macrocycles (e.g., **38**) in good yield and high stereoretention (Scheme 6b).

**Scheme 7:**
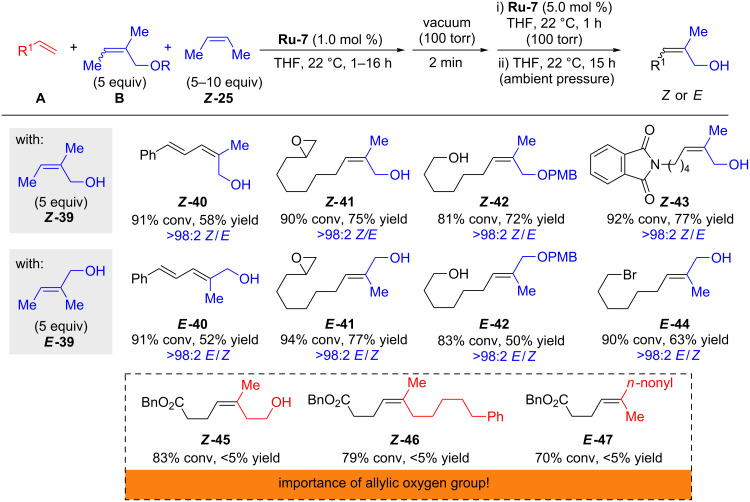
Cross metathesis with *Z*- and *E*-trisubstituted allylic alcohols.

More recently Hoveyda disclosed his findings concerning the synthesis of *Z*- or *E-*trisubstituted allylic alcohols with ruthenium dithiolate catalysts (Scheme 7) [22]. In agreement with the proposed model (Figure 3), **Ru-7** was significantly more efficient compared to **Ru-3**. The reason for the higher reactivity of *E*-stereoretentive catalysts with trisubstituted substrates was previously discussed in the section "Mechanistic models". Cross metathesis utilizing the in situ methylene capping strategy with 1,1-disubstituted allylic alcohols ***Z*-39** or ***E*-39** afforded the products **40**–**42** in good yield and with excellent retention of stereochemistry independent of the configuration of the allylic alcohol. Allylic oxygen atoms often have an activating effect in metathesis [23]. This was confirmed by Hoveyda for stereoretentive metathesis by exposing homoallylic alcohol (product ***Z*-45**) and alkyl containing metathesis partners (products ***Z*-46** and ***E*-47**) to standard reaction conditions [22]. All the reactions were inefficient emphasizing the importance of an allylic alcohol, ether or acetate group.

### 7 The in situ catalyst synthesis strategy

**Scheme 8:**
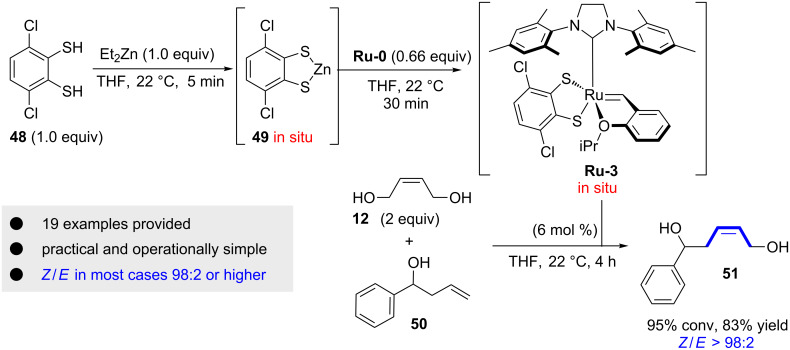
In situ synthesis of **Ru-3** and application thereof in the cross-metathesis of **12** and **50**.

Very recently, our group developed an in situ synthesis of dithiolate catalysts with the aim to avoid tedious isolation of Ru-dithiolate catalysts and to render this class of catalyst available to every practicing chemist [25]. A very practical and operationally simple protocol for the in situ generation of Ru-dithiolate catalysts was reported. First, the commercially available dithiol **48** is deprotonated with Et_2_Zn to provide Zn-dithiolate **49** (Scheme 8). Then Hoveyda–Grubbs catalyst **Ru-0** is added to generate after another 30 minutes a solution of the desired catalyst **Ru-3**. Finally, the ruthenium stock solution of **Ru-3** is added to the alkene starting material (e.g., for the cross metathesis of **12** and **50**) to give the product in high yields and excellent stereochemical purity. We applied the in situ generated catalyst to several reactions including cross metathesis, self-metathesis and RCM reactions. The selectivities are in general very high (*Z*/*E* = 98:2 or higher).

### 8 Applications in the synthesis of biologically active compounds

**Figure 6:**
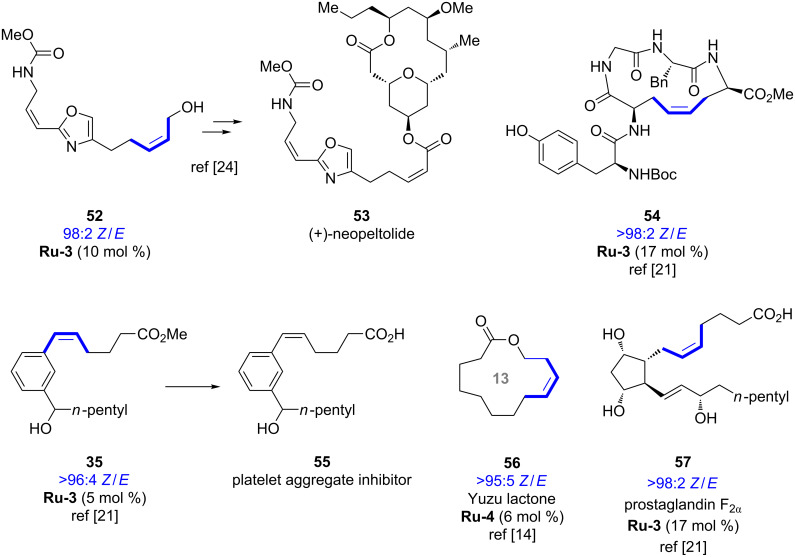
Examples of biologically active and fragrance molecules synthesized by stereoretentive metathesis.

Several biologically active compounds, fragrance molecules and natural products were synthesized utilizing stereoretentive metathesis based on Ru-dithiolate catalysts, for example (+)-neopeltolide (**53**, Figure 6) [24]. For each of the examples the catalyst loading of the Ru-dithiolate catalyst which was required to forge the corresponding *Z*-olefin is indicated. Given the high stereoisomeric purity of the obtained products we can expect many other examples to be reported in the near future.

## Conclusion

Within only a few years the field of stereoretentive metathesis using ruthenium dithiolate catalysts has attained a remarkable level of maturity. The fast development in this field is due to the complementary contributions of the Hoveyda and Grubbs groups who developed a set of general and highly stereoretentive Ru-dithiolate catalysts. A major limitation at the moment is that the *Z*-stereoretentive method is much more efficient and practical compared to stereoretentive methods for *E*-alkenes. Certainly making the precursors of **Ru-6**, **Ru-7**, **Ru-8** and **Ru-9** commercially available would significantly help to further promote *E*-stereoretentive metathesis. Nevertheless, it can be stated that the field has come a long way compared to where it was 5 years ago and certainly further important improvements will be reported in the near future.
